# Laparoscopic appendicectomy without the use of disposable materials - a low-cost alternative - 1,552 cases operated in 20 years

**DOI:** 10.1590/0100-6991e-20222446

**Published:** 2022-03-11

**Authors:** CARLOS EDUARDO DOMENE, PAULA VOLPE, ANDRÉ VALENTE SANTANA

**Affiliations:** 1 - Centro Integrado de Medicina Avançada e Núcleo Unificado de Tratamento do Obeso, Cirurgia - São Paulo - SP - Brasil

**Keywords:** Appendectomy, Laparoscopy, General Surgery, Apendicectomia, Laparoscopia, Cirurgia Geral

## Abstract

**Introduction:**

Laparoscopic appendectomy does not have a single protocol on its technical systematization, access routes, and use of energy and staplers. The cost of disposable materials can prevent its widespread use. Alternatives to decrease cost can help disseminate the laparoscopic access to appendectomy.

**Objective:**

to introduce a low-cost laparoscopic appendectomy method with good aesthetic results through the location of incisions; to show its viability through its application in 1,552 cases of laparoscopic appendectomy operated between 2000 and 2019 with three portals and very low-cost regarding materials used.

**Methods:**

we applied three punctures - an umbilical one for the camera (5 or 10mm in diameter), a 10mm puncture in the right iliac fossa, and one 5mm puncture in the left iliac fossa. The materials used were permanent use trocars, grasping forceps, hook, scissors, and needle holder, without the need for any disposable device.

**Results:**

1.552 patients were operated between 2000 and 2019, 56.2% being female, mean age 32.66 years (9-93), average hospital stay of 1.74 days (1-10), and median of 1.2 days.

**Conclusion:**

the technique we describe uses three metallic trocars and four permanent instruments, in addition to a single cotton suture. It is, therefore, a very low-cost laparoscopic procedure. Its application has shown good results and low morbidity, which may become the preferred indication for laparoscopic surgery in the treatment of acute appendicitis.

## RESUMO

**Introdução:**

a apendicectomia videolaparoscópica não tem protocolo único sobre sistematização técnica, vias de acesso, uso de energia e grampeadores. O custo de materiais descartáveis pode inviabilizar o emprego mais generalizado. Alternativas para diminuir o custo podem ajudar a disseminar o acesso laparoscópico para a realização de apendicectomia.

**Objetivo:**

introduzir um método para realizar a apendicectomia videolaparoscópica de baixo custo e visando bom resultado estético por meio da localização das incisões; mostrar a viabilidade por meio de aplicação em 1.552 casos de apendicectomia videolaparoscópica operados entre 2000 e 2019 com três portais, de muito baixo custo em insumos utilizados.

**Métodos:**

três punções - uma punção umbilical para introdução da câmera (de 5 ou 10mm de diâmetro), uma punção de 10mm em fossa ilíaca direita e uma punção em fossa ilíaca esquerda de 5mm. Os materiais utilizados - trocartes, pinças de apreensão, gancho, tesoura e porta-agulhas são de uso permanente, sem necessidade de qualquer material descartável.

**Resultados:**

foram operados 1.552 pacientes entre 2000 e 2019, sendo 56,25% do sexo feminino, média de idade de 32,66 anos (9 a 93 anos), tempo médio de internação de 1,74 dias (1 a 10 dias) e mediana de 1,2 dias.

**Conclusão:**

a técnica que descrevemos utiliza três trocartes metálicos e quatro instrumentos permanentes, além de um único fio de algodão. Trata-se, portanto, de procedimento laparoscópico de muito baixo custo. A aplicação demonstrou bons resultados e baixa morbidade, podendo tornar-se rotina a indicação preferencial da vídeocirurgia no tratamento da apendicite aguda.

## INTRODUCTION

Appendectomy is the most frequent operation in emergency situations^1^ ^-^ ^4^.

Despite the record of more than 30 years since the first laparoscopic appendectomy, it is still performed by laparotomy in at least two thirds of cases^1^ ^-^ ^5^.

Several causes determine this high rate of laparotomic procedures in this disease, among them:

1. cost of equipment and supplies used; and
2. lack of systematization that dispenses with disposable and high-cost instruments, such as disposable trocars, staplers, bipolar or ultrasonic energy forceps, and disposable specimen extractor bags.

We describe a laparoscopic appendectomy technique with three portals, with very low cost in terms of used materials. The incisions allow minimizing the exposure of scars, as long as they are located in the umbilicus and iliac fossas. The materials used are of permanent use, dispensing with any high-cost disposable device. We demonstrated the feasibility of this technique by using it in 1,552 patients over a 20-year period.

## METHODS

We perform the first puncture in the umbilicus, 5 or 10mm in diameter, for the placement of the endoscope (depending on its availability), with a permanent metallic trocar. In the postoperative period, this incision is imperceptible because it is located inside the umbilicus.

We add two punctures in a bilateral low pelvic position, medially to the epigastric vessels.

On the right side, we introduce a 5mm diameter, permanent, metallic trocar; on the left, we insert a permanent, metallic trocar with a diameter of 10mm and a reducer to 5mm. These punctures are also imperceptible, as they are hidden below the patient’s underwear line.

**Figure 1:**
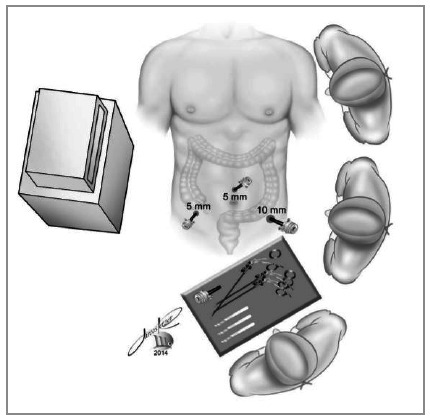
Positioning of the surgical team, punctures and monitor.

The surgeon stands on the patient’s left, with the assistant on the right and the scrubs table on the left. The monitor is located to the patient’s right (Figure 1).

The operation is performed with four permanent instruments: grasper forceps, hook, scissors, and needle holder. We use a single threaded 2-0 cotton suture.

**Figure 2:**
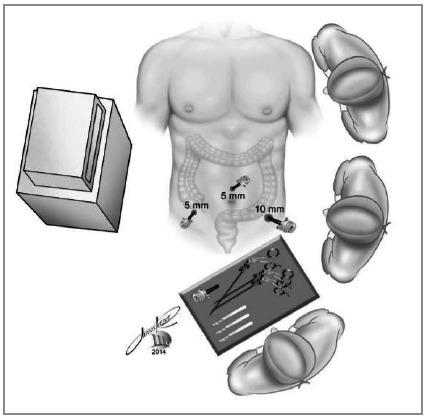
Release of the meso appendix with monopolar hook.

**Figure 3:**
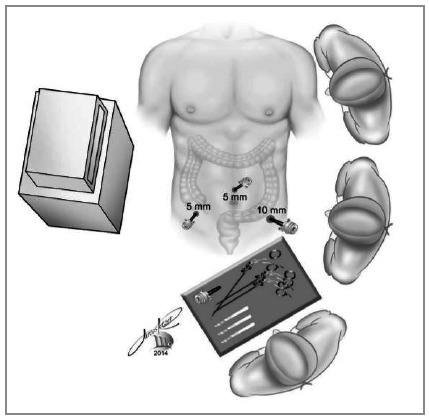
Appendix section near the ligature with the monopolar hook.

**Figure 4:**
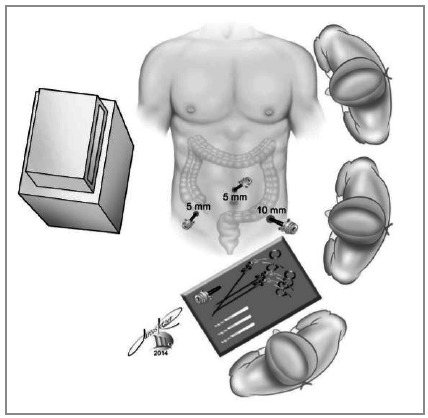
Traction and removal of the appendix through the left iliac fossa trocar immediately after its section, avoiding use of an extractor bag.

The operative technique consists of the following steps:

1. apprehension of the cecal appendix with the grasper introduced through the right iliac fossa.
2. with the hook on the left iliac fossa trocar, the appendix is progressively isolated from its mesentery, from end to base. The cecum near the appendix is released from epiploic appendages that are located near the appendicular base (Figure 2).
3. we suture the base of the appendix with a 20cm threaded 2-0 cotton suture, transfixing the serosa in two points for better fixation of the ligature. Another more distal suture is optional for sectioning the appendix between sutures, without risk of content leakage. Section of the suture thread; the remainder of the suture stays in the abdominal cavity for future realization of the appendicular stump invagination pouch.
4. grasping of the appendix close to the base (with the grasper introduced with a reducer in the 10mm trocar of the left iliac fossa), or between the two sutures, when the second suture was performed (Figure 3).
5. Section of the appendix using the hook introduced through the right iliac fossa, between the base suture and the grasper grip (or between the two sutures performed), avoiding extravasation of the appendix contents (Figure 3).
6. Removal of the apprehended appendix, pulling the grasper immediately after the section, into the trocar (Figure 4).

In most cases, the diameter of the appendix without the mesentery allows for removal of almost all specimens, even very inflamed ones, by pulling the grasper and reducer inside the 10mm trocar.

7. The appendix is pulled and placed inside the 10mm trocar.
8. The 10mm trocar is removed from the abdominal wall with the appendix inside it. After exiting the appendix, the trocar is again introduced into the abdominal wall.

This maneuver avoids the use of extraction bags, which, in addition to increasing the cost of the procedure, requires maneuvers to introduce the appendix into their interior, which can be time-consuming and risk contaminating the abdominal cavity.

Pouch suture in the cecum around the appendicular stump for invagination.

## RESULTS

 A total of 1,552 patients underwent surgery between 2000 and 2019, 56.3% of whom were female, with a mean age of 32.7 years (9-93).

Patients had appendicitis at all stages of evolution (Table 1) - from edematous to purulent to necrotic.

**Table 1:** 

Appendicitis phases	Number of patients	Percentage (%)
Edematous	821	52.9%
Purulent	488	31.4%
Necrotic	242	15.6%

In four patients, appendiceal necrosis extended to the cecum, with inflammation and perforation in two of them, requiring a right colectomy, also completed by a totally laparoscopic approach.

There were no conversions to open procedure or the need to insert additional trocars to perform the procedure, except in the cases of modification of the conduct for right colectomy.

In all cases, it was possible to inspect the entire abdominal cavity and irrigate and aspirate all abdominal quadrants through the access used when there were purulent collections.

We drained 84 cases in which there was an abscess in the right iliac fossa, using a silicone Penrose drain.

Antibiotic prophylaxis was used in cases of edematous appendicitis with a 2^nd^ generation cephalosporin; in the other patients, we administered antibiotic therapy with 3^rd^ generation cephalosporin and nitroimidazole for 7 days.

The mean length of hospital stay was 1.74 days (1-12), median of 1.2 days. There were six readmissions in the first 30 days, with significant abdominal pain or fever, and in two there was need for relaparoscopy for aspiration of a pelvic purulent collection. Hyperemia and inflammation of the left iliac fossa incision occurred in 74 cases (4.7%), but there was no case of abscess that required drainage in the incisions performed.

There was no mortality. 

## DISCUSSION

Described by Fitz and performed for the first time in 1886 for the treatment of acute appendicitis, appendectomy is the safest treatment for this condition at any stage of its evolution6. The incisions used for laparotomic access vary widely, but the most common is the access proposed by McBurney (oblique incision in the right iliac fossa)^7^. The aesthetic result is quite precarious when using laparotomy incisions, whether oblique, horizontal, or vertical. Most appendectomies are performed in children and adolescents, and esthetics is an extremely important factor in the evaluation of the appendix extraction method^8^. These scars will remain for life, and may change as the patient grows, often becoming very unsatisfactory.

The introduction of the laparoscopic approach for appendectomy, described by Kurt Semm in 1982^1^, brought significant aesthetic benefits, since it is almost always performed with three punctures, two of which in different positions on the abdominal wall, but which, depending on the location, can be visible when the abdomen is exposed. This is of particular importance when the operation is performed on female adolescents.

Access through natural orifices (excluding the umbilicus from this classification) - NOTES - has not had a significant evolution and, even if it can be used in the future, it will require instruments and specialized equipment, increasing the cost of the procedure, in addition to requiring a highly trained team to carry it out^9^.

The single umbilical access technique is feasible for appendectomy and has better aesthetic appeal compared with multiple visible incisions in the anterior abdominal wall^10^ ^-^ ^17^.

For single access, the incision needs to be larger, and it can become visible or deform the patient’s umbilicus. The incision must be at least 2.5cm long for placement of a special trocar or three conventional trocars^8^ ^,^ ^18^ ^,^ ^19^. Most indications for appendectomy are in the age group of children, adolescents, and young adults. An incision of this size can determine a very poor aesthetic result in these cases^20^ ^,^ ^21^. The descriptions of single access case series show that an additional auxiliary trocar may be necessary in up to 10% of cases^22^, or even be converted to the three-trocar technique, compromising the esthetic aspect^12^. In comparative studies, there were complaints of more intense pain compared with conventional laparoscopy^23^ ^,^ ^24^. The cosmetic result was considered better or equal to the conventional method^9^ ^,^ ^16^.

Even if it is superior in terms of aesthetic results than conventional laparoscopy, the cost can be significantly higher, due to the need to use special devices to introduce the instruments through a single access^6^ ^,^ ^21^ ^,^ ^22^. To reduce costs, placement of three 10 and 5mm diameter trocars together can be performed through a single enlarged umbilical incision^25^ ^,^ ^26^; descriptions of this tactic have led authors, in comparative studies, to consider the performance difficult and the final aesthetic appearance similar to laparoscopy with three portals^12^ ^,^ ^13^ ^,^ ^20^.

In the single-port technique, the technical difficulty of performing the dissection and section of the appendix with conventional instruments without triangulation and in a poor position to visualize the operative field cannot be disregarded, rendering the procedure riskier and hampering its use in cases where the appendices are more difficult to resect^27^ ^,^ ^28^.

Safety is an important aspect to be considered in operations as common as appendectomy, in which even the number of procedures performed by each surgeon has an implication in increased complications, length of stay, and costs^29^ ^,^ ^30^. On the other hand, if performed safely and using a minimally invasive method, in a specialized environment, discharge rates can be achieved in less than one day in up to 90% of cases^30^ ^,^ ^31^.

The technique described in this article, with three portals - one umbilical and two in the left and right lower quadrants (medially or laterally to the epigastric artery) - has a different aesthetic result than when making lateral incisions in the abdomen, as the scars remain below the underwear line when the abdominal wall is exposed. The umbilical incision scar is small, and it can even be 5mm, not causing deformation of the umbilicus in any of the patients. The other two incisions remain under the underwear, determining that, in most cases, scars are not visible or identifiable when the abdomen is exposed. These characteristics may be important when considering that young females are operated on in a tropical country where the abdomen is more exposed, and patients are satisfied with this incision’s arrangement.

This technique also enables appendectomy with a privileged view of the appendix and of the operative instruments. It allows adequate triangulation of instruments, bringing safety and shorter operative time for the procedure. The use of very close trocars can make triangulation difficult^32^. In the technique described, the trocars are in the iliac fossae, far enough apart to allow adequate triangulation.

The treatment of the meso-appendix, appendix, and appendicular stump is the subject of many publications^12^ ^,^ ^33^.

In releasing the meso appendix, the use of endoclips, bipolar forceps, harmonic forceps, or staplers with vascular loads are described. In all, or almost all of these tactics, the meso appendix remains next to the appendix, greatly increasing the volume of the surgical specimen, forcing extraction in special bags, which increase costs and operative time^31^ ^,^ ^32^. It should be noted that the removal of the meso appendix is a mere operative tactic, as it can remain close to the cecum without any additional risk.

Furthermore, by dissecting the mesentery next to the appendix, the technique described makes ligation of the appendicular artery unnecessary, avoiding the use of devices for its occlusion and reducing the risk of bleeding. We used monopolar energy at 30% of the maximum level, dissecting the meso appendix next to appendix, where the vessels have smaller caliber. This way, the release of the appendix does not determine significant intraoperative bleeding. There was no need for reintervention due to bleeding in any of our series.

The surgical specimen, being only the inflamed appendix, can be removed in almost all cases inside the 10mm trocar of the left iliac fossa, reducing the risk of contamination of the abdominal wall during extraction (Figure 4).

If it is not possible to remove the appendix through the trocar, we use the artifice of introducing a small sterile plastic bag or a piece of very low-cost surgical glove, removed through the incision of the left iliac fossa.

The appendicular stump can just be sutured with 2-0 cotton suture and left exposed. Comparative studies between simple ligation and invagination do not show differences between the two methods of treatment of the appendiceal stump^33^. In the technique described, we performed pouch-string sutures in the cecum around the appendicular stump and invagination.

This procedure, being standardized and simplified, can be performed in any hospital environment that has a conventional laparoscopy system with basic permanent forceps, and makes the operation faster, removing the specimen quickly, and with only one or two sutures. As it does not use any disposable instruments or devices, it is very low cost^34^ ^,^ ^35^. It can be used safely in any age group, at any stage of appendicitis evolution, especially in morbidly obese patients^36^ ^,^ ^37^.

We have used this technique systematically in the last twenty years, in 1,552 operated patients, with low morbidity and no mortality. Access through three portals made it possible in all cases to complete the surgical resolution of the case, without the need for an additional trocar, attesting to safety, efficiency, and reproducibility. We hope with this contribution to make it possible for laparoscopic appendectomy to be more widely applied in the surgical treatment of acute appendicitis, since there is already concrete evidence on its superiority over laparotomy appendectomy, especially in more complicated cases or in obese patients.

This article has several limitations. This is a retrospective study and the results do not analyze all aspects related to the patients’ evolution. Its purpose is to demonstrate that laparoscopic appendectomy can be performed at low cost without the use of disposable material and shows the safety of the application of this systematization.

## CONCLUSION

The technique we describe uses three metallic trocars and four permanent instruments, in addition to a single cotton suture. It eliminates the use of surgical specimen extraction bags, clips, straps, staplers, or special energy, bipolar, or harmonic instruments. It is, therefore, a very low-cost laparoscopic procedure. As it allows triangulation and instrumentation in the conventional way, it is a highly safe and reproducible operation, can be easily taught, and have its use multiplied in hospitals that have conventional laparoscopic equipment.

The results of 1,552 operated patients, with low morbidity and no mortality, attest to the safety and reproducibility of the described approach.
